# Vehicle Vibration Reduction Using Hydraulic Dampers with Piezoelectric Valves

**DOI:** 10.3390/s25041156

**Published:** 2025-02-13

**Authors:** Lech Knap, Cezary Graczykowski, Jan Holnicki-Szulc

**Affiliations:** 1 Institute of Vehicles and Construction Machinery Engineering, Warsaw University of Technology, Narbutta 84, 02-524 Warsaw, Poland; lech.knap@pw.edu.pl; 2 Institute of Fundamental Technological Research, Polish Academy of Sciences, Pawińskiego 5B, 02-106 Warsaw, Poland; holnicki@ippt.pan.pl

**Keywords:** semi-active suspension, piezoelectric valve, suspension sensors, ride comfort and safety

## Abstract

Ensuring adequate comfort and safety in vehicle motion is a subject of extensive research worldwide. Despite the implementation of new control algorithms, including those leveraging AI, the application of effective semi-active vibration dampers remains crucial for achieving optimal suspension performance. This article presents experimental studies conducted on a vehicle equipped with semi-active suspension featuring custom-designed hydraulic dampers controlled by piezoelectric valves. These innovative dampers are characterized by extremely short response times, enabling real-time adaptation to varying driving conditions. A simple control algorithm designed to operate based on real-time signals from suspension sensors is introduced and evaluated. The experimental setup, including the measurement system used during testing, is described in detail. The presented results highlight the significant potential of this approach for improving driver comfort under specific driving conditions, even without detecting road roughness ahead of the vehicle.

## 1. Introduction

Ensuring adequate ride comfort and passenger safety has long been a challenge in the design of suspension systems. Significant advancements in electronics, the development of control technologies and algorithms, and progress in materials science over the past several decades have enabled the introduction of modern solutions in the form of so-called semi-active or active suspension systems. These systems offer a substantial improvement in the dynamic characteristics of vehicles compared to traditional passive suspensions [1,2,3]. Traditional passive suspensions rely on two primary components: springs, which provide the necessary suspension stiffness, and dampers with fixed characteristics responsible for energy dissipation. While such systems are relatively simple and cheap and perform adequately in many driving scenarios, they can also lead to excessive driver and passenger fatigue or contribute to hazardous situations on the road under certain conditions. From a comfort perspective, issues may arise due to the excessive intensity of accelerations affecting the occupants [4]. Regarding safety, variations in the normal forces between the tire and the road surface can result in a loss of traction or wheel slip, which may lead to dangerous driving conditions [5,6].

The introduction of semi-active suspensions with controllable dampers that adapt their characteristics in real time allows dynamic adjustment of suspension parameters, e.g., damping forces, to current road conditions, rather than relying on pre-defined settings optimized for the most frequent scenarios, as in the case with passive suspensions. However, the effective operation of such semi-active suspension systems requires not only the development of controllable dampers but also the creation of advanced control algorithms and a network of sensors capable of monitoring immediate and extended environmental parameters affecting the vehicle [7].

Semi-active solutions are based on various types of controllable dampers, whose properties are adjusted through different types of valves or electrically controlled elements that restrict the flow of oil or air between the working chambers of the damper [8]. For instance, the authors of [9] presented the results of research and modeling of a damper equipped with an electromagnetic CCD valve, while [10] introduced the concept of a hydro-pneumatic suspension controlled by proportional valves. In [11,12], the authors presented experimental studies and laboratory and vehicle models of a hydraulic damper controlled by a valve with a piezoelectric actuator. A similar solution employing a piezoelectrically actuated valve was applied in a pneumatic damper, which demonstrated comparable performance and was designed to protect aircraft during landing [13]. An interesting extension of piezoelectric material applications includes attempts to use piezoelectric actuators to construct dampers capable of recovering energy from the vehicle body vibrations as presented in [14,15,16].

At the beginning of the century, significant expectations were placed on the development of magnetorheological fluid (MRF)-based dampers. These devices were extensively reviewed in [17,18]. Despite the passage of time, research into MRF-based dampers continues, focusing on their construction, operation, and numerical modeling [19,20,21]. Other studies in [22,23] presented experimental findings on MRF dampers, their numerical models, and proposed control algorithms optimized regarding comfort or safety criteria. A new generation of MRF dampers, rotary motion dampers, has recently gained considerable attention in research [24]. Efforts have also been made to utilize electrorheological fluids (ERFs) in damper construction [25], though with limited success. Other researchers have explored the development of novel damper technologies with alternative operating principles, such as friction dampers [26] or those based on the controlled motion of particles located inside a compartment of controllable size [27]. These ongoing studies demonstrate the breadth of innovation in the field of controllable dampers, underscoring their critical role in advancing semi-active and active suspension technologies.

A semi-active vehicle suspension system, on which we concentrate in this article, is a prime example of a cyber-physical system (CPS), characterized by a complex architecture that integrates a physical and a virtual layer. The physical layer collects signals from sensors and controls actuators (dampers), while the virtual layer executes the developed control algorithm [28]. Upon receiving environmental parameters from the sensors, the control algorithm determines the actuator signals based on a vehicle model or a subset thereof. Given this complexity, a significant portion of research on semi-active vehicle suspensions focuses on control units, algorithms, and the selection of sensors necessary to ensure proper operation under actual conditions [29,30]. For vehicle suspensions, two primary control approaches are typically employed. The first focuses on minimizing accelerations (referred to as the comfort criterion), while the second targets are maintaining stable values of contact forces between the tire and the road surface (the safety criterion). These criteria conflict, leading researchers to seek compromises between them [31,32]. A practical solution to the control problem was proposed in [33], where the control signals were derived using a 7-degree-of-freedom vehicle model, leveraging LiDAR technology for continuous road profile and vehicle height data acquisition. A similar approach was presented and discussed in [34]. Other extensive simulation studies of a complete vehicle model were detailed in [35]. A distinctly different method based on pressure sensors was introduced in [36,37], where the authors demonstrated several effective variations in predictive control algorithms for pneumatic dampers and tested them experimentally at a drop-test stand [38,39]. Also, neural networks and machine learning techniques are increasingly employed to address challenges in suspension control [40,41]. An alternative approach to mentioned-above solutions was suggested in [42], where the authors sought to simplify the system by utilizing only a single accelerometer to ensure the effective operation of their control algorithm.

All the above advancements underscore the diverse strategies being explored to optimize semi-active suspension systems, highlighting the interplay between sensor technology, algorithm development, and vehicle modeling in achieving enhanced ride comfort and safety. It is evident that intensive research efforts continue to focus on developing new generations of semi-active vehicle suspension systems. While some researchers employ increasingly sophisticated tools to design advanced control algorithms, others aim to develop simple, fast, and effective algorithms that rely on single-sensor inputs.

While semi-active fluid-based suspensions offer significant advantages over passive systems, they have inherent limitations, which result from the applied control strategies, the physical constraints of the control system, and the dynamics of the vehicle. As highlighted in [43], commonly applied control strategies for vibration isolation include skyhook control [44], groundhook control [45], and acceleration-driven damper control [46]. Each of these control strategies exhibits the best performance within specific frequency ranges. Specifically, the skyhook control demonstrates very good vibration isolation capabilities at low frequencies near vehicle body resonance, while acceleration-driven damper control is the most effective at high frequencies near wheel vibration resonance. To address these limitations, attention has been directed towards combining these control strategies. Notably, mixed strategies, such as those integrating skyhook control with acceleration-driven damper control [47], and approaches combining skyhook and groundhook control [48], have proven to be effective across a much broader frequency spectrum. However, a significant drawback of these combined methods is the increased complexity of the control algorithms and difficulties in their application in realistic scenarios where excitation frequency continuously changes. Due to the aforementioned limitations of existing semi-active suspension systems, there still remains a significant need for the development of novel vibration isolation systems and control strategies, which provide their efficient operation. The semi-active hydraulic damper equipped with a piezoelectric valve and the control system proposed in this study are one of them attempts to resolve the above research challenge.

In this study, the authors proposed a straightforward approach to designing a semi-active vehicle suspension system. The suspension system utilizes two custom-designed controllable dampers featuring piezoelectric actuator valves (PZD-FT). These dampers enable rapid adjustment of generated damping forces based on the identified quite wide range of damping characteristics. In addition, the study introduces a simple and fast control algorithm based on a single-wheel vehicle model, relying predominantly on a single type of sensor for suspension deflection measurement. This control algorithm was experimentally validated on a vehicle equipped with PZD-FT dampers. The archived and presented results demonstrate that, at low and medium speeds, the proposed system can significantly enhance driver and passenger comfort by increasing the so-called exposure time up to approximately 136%. As a result, the system enables extended travel durations under more comfortable conditions.

The primary advantages of the proposed system stem from both the proposed straightforward system design and the proposed online control method. Firstly, the system design integrates the hydraulic damper with an additional bypass and piezoelectric valve for control of the hydraulic fluid flow. This configuration can be seamlessly incorporated into standard passive hydro-pneumatic suspensions, making it highly adaptable to a wide range of passenger vehicles. Secondly, the proposed online control method requires only a single input signal (real-time suspension deflection) and employs a simple semi-analytical method for determining the control signal and the optimal valve opening providing efficient damper operation. This results in exceptional system simplicity and reliability due to independence of complex sensor systems and detailed vehicle dynamics models, which change over time due to the degradation of suspension components and are often sensitive to disturbances. Both of the above features underscore the practical significance of the proposed semi-active suspension and strong potential of its widespread application.

The study is organized as follows: Section 2 provides a concise overview of the design and characteristics of the PZD-FT damper. Section 3 outlines the employed control algorithm, derived from a simplified version of more advanced control strategies. Section 4 presents the measurement system and the applied sensors, which are essential for the proper functioning of the vehicle suspension system. Section 5 focuses on road tests and the obtained results. The paper concludes with a summary and key findings.

## 2. Hydraulic Damper Controlled by Piezoelectric Valve

The design of the PZD-FT damper, controlled by a piezoelectric valve and intended for installation in a laboratory Ford Transit vehicle, largely relies on the use of the factory damper construction. The original Ford Transit damper belongs to the group of so-called twin-tube shock absorbers, featuring two valves: (i) a base valve located between the chamber below the piston and the chamber in the casing, and (ii) a piston valve separating the working chambers. During compression (jounce), the space beneath the piston decreases, necessitating the displacement of oil to other working chambers. The oil flows through the base valve and piston valve to balance the volume in the upper chamber above the piston (which slightly increases in volume despite being partially occupied by the piston rod). In this mode, the damper’s characteristics are primarily determined by the base valve. In the case of rebound, the situation is reversed. Oil is drawn from the oil reservoir into the chamber below the piston through the base valve, which offers minimal resistance during this flow. Meanwhile, the oil in the upper chamber above the piston is compressed, and its flow through the piston valve governs the damper’s characteristics.

**Figure 1:**
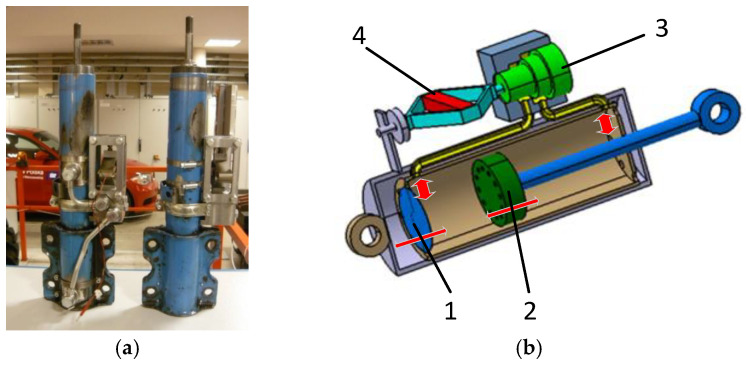
PZD-FT damper: (**a**) PZD-FT damper based on OEM damper design, (**b**) PZD-FT damper diagram: 1—bottom valve, 2—piston with valves, 3—PZV valve, 4—piezoelectric actuator [12].

**Figure 2:**
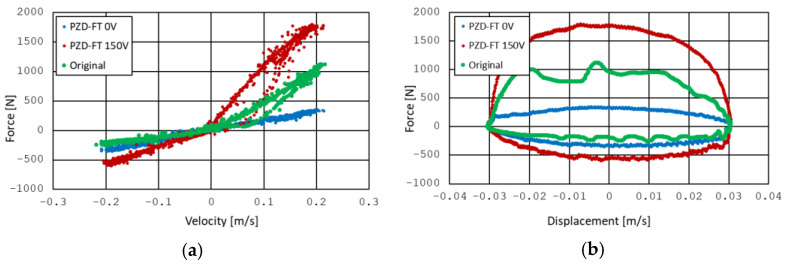
Experimental damping characteristics of PZD damper on (**a**) velocity–force plane and (**b**) displacement–force plane. PZD-FT 0 V (blue line)—PZD valve without supplied voltage (0 V), PZD-FT 150 V (red line)—PZD valve with supplied voltage (150 V), original (green line)—factory passive damper [12].

The modified factory shock absorber is shown in Figure 1a, and its construction schematic is illustrated in Figure 1b. As evident, the PZD-FT damper was created by adding a bypass connection between the chambers on both sides of the piston (2). Installed on this bypass is a valve (3) controlled by a piezoelectric actuator (PZD), APA-120L, manufactured by Cedrat (4) [49]. The PZD valve regulates the flow of oil between the chambers on either side of the piston by adjusting the size of the gap through which the oil flows. This gap adjustment is achieved by varying the voltage supplied to the piezoelectric actuator within a range of 0–150 V. This configuration, combined with the partially functional factory-installed valve inside the piston, allows for shaping the damper’s dissipation characteristics and extending the factory-provided performance curve. The dissipation characteristics of the PZD-FT damper, derived from experimental tests, are presented in Figure 2. The characteristics shown in Figure 2 were obtained experimentally on the test bench with the use of kinematic distortion. During the tests, the dampers were excited kinematically at different amplitudes and frequencies. Displacements of the damper rod and corresponding force were recorded during the tests. By varying the supply voltage signal, it was possible to obtain the characteristics of the PZD-FT damper for different voltages in the range of 0–150 V. A more detailed description of the PZD-FT damper’s construction, experimental test bench, results, and rheological model identification can be found in [12].

The characteristics presented in Figure 2 show the results for kinematic excitation performed at a frequency of 1 Hz with an amplitude of 30 mm. The characteristics demonstrate that by applying the PZD valve, it was possible to extend the range of forces achievable compared to the original factory damper. It is also evident that the damper’s characteristic is asymmetric, i.e., different during the compression and extension of the damper. It is also worth noting that the experimental results [12] indicated that the damper exhibits a fast response time, allowing the control characteristics to change in approximately 9–10 ms.

The obtained results indicate linear properties of the PZD-FT damper, with the dominant role of damping behavior alone. The impact of the elastic elements and friction properties is limited, and there is no need to model the PZD-FT damper’s characteristics using more complex models, such as Herschel–Bulkley [50], Casson [51], or Bingham [52].

**Figure 3:**
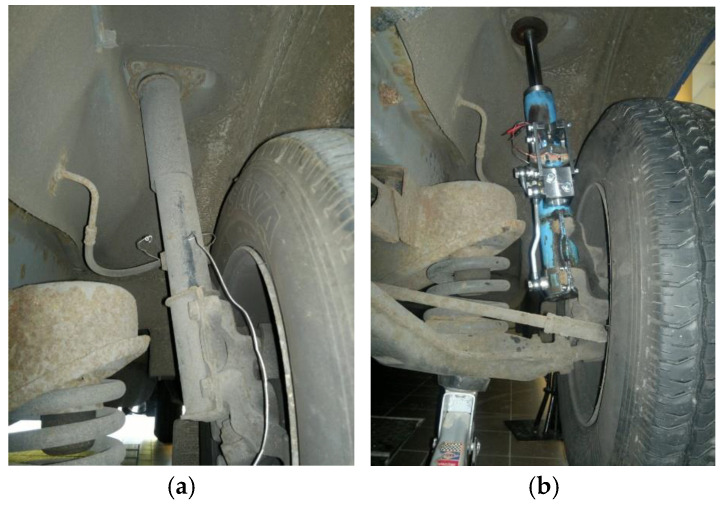
Views of the front wheel suspension of the Ford Transit: (**a**) original suspension, (**b**) suspension with the PZD-FT damper [12].

One of the reasons for using the original damper in the construction of the PZD-FT damper was the need to ensure safety during driving. The original damper housing ensures the proper functioning of the MacPherson strut, which is used in the front suspension of the Ford Transit. After experimental testing in the laboratory, the developed PZD-FT dampers for the front suspension were installed on the laboratory vehicle Ford Transit, as shown in Figure 3. In the rear suspension of the vehicle, the original dampers were remained unchanged.

## 3. Vehicle Suspension Control Algorithm

The key role of the control algorithm is to determine the appropriate control signal, i.e., the voltage required to power the PZD-FT dampers. By selecting the correct control signal during operation, it is possible to shape the damping force T in such a way that the desired control criterion is met. The control algorithm, outlined below in simplified form, is based on the assumption that limiting the variation in forces acting on the vehicle body, which originate from the suspension (i.e., the sum of the damping and spring forces for each wheel), will result in a reduction in the vertical accelerations acting on the vehicle body [23].

**Figure 4:**
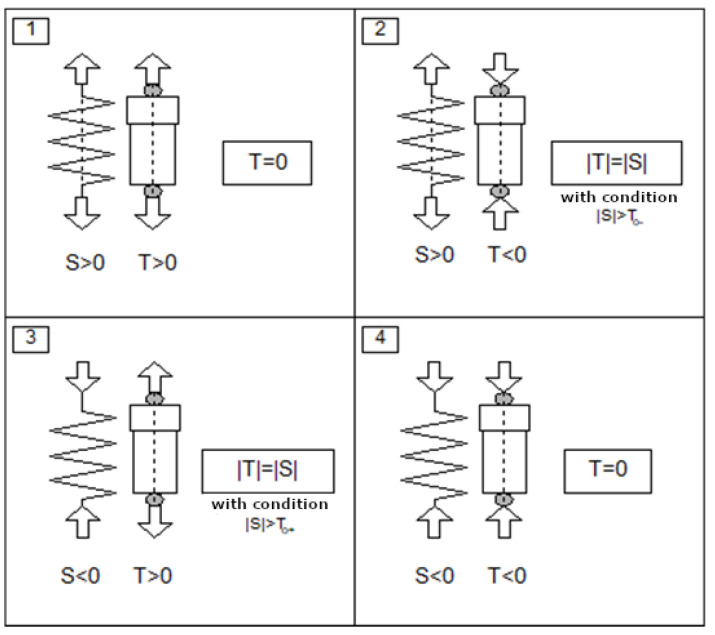
Methods for determining the required damping force *T* based on algorithm that minimizes accelerations in the suspension of the tested laboratory Ford Transit vehicle, corresponding to four different cases of vehicle suspension operation (1–4).

Figure 4 presents a diagram of how the control algorithm determines the value of the damping force of the PZD-FT damper to meet the criterion of minimizing vertical accelerations, specifically in relation to a single wheel of the vehicle. In this case, minimization is possible when, at any given moment, the sum of the elastic forces S (originating from the suspension’s elastic elements) and the damping forces T generated by the PZD-FT dampers is minimized. This relationship can be described by the following expression:

(1) $$\underset{T}{\min}\left|S+T\right|,$$

From this expression, it follows that:

- In the case where the elastic force S has the same direction as the damping force T in the damper, the optimal value of the damping force should be its minimum value, i.e., $T=T_{min}$;
- In the case where the elastic force S has the opposite direction to the damping force T in the damper, the optimal value of the damping force should be equal to the elastic force S, i.e., T=S, with the constraint that $T\in \left[T_{min},T_{max}\right]$.

The above cases are separated into four distinct situations and they are presented in Figure 4 as cases 1–4. The arrows next to the elastic and damping components indicate the directions of the forces exerted by these elements on the vehicle (suspension components) at different moments of suspension operation. The directions of the forces shown in Figure 4 depend on the suspension deflection and its velocity. It is necessary to refer to the initial position of the suspension deflection, which corresponds to the static deflection of the vehicle suspension under the influence of the vehicle’s total weight, including the load and the number of passengers. For example, such deflection identification can be easily achieved when the vehicle is started.

The spring force S(t) in Expression (4) at any given moment in time is determined based on the current measurement of the suspension deflection u(t) and the known linearized value of the suspension stiffness constant k:

(2) $$S\left(t\right)=ku(t),$$

**Figure 5:**
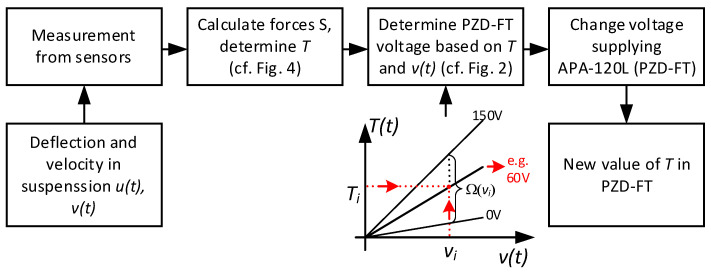
A block diagram algorithm for selecting the control voltage for the PZD-FT dampers in the research Ford Transit vehicle.

The value of the force $T\left(t\right)$ at a given moment can be determined based on the relationship in Equation (1), as also illustrated in Figure 4. Calculating this force value is then used to determine the control signal, i.e., the calculation of the control voltage for the operation of the PZD-FT damper. The control voltage is derived based on the similar characteristic shown in simplified form in Figure 4 (cf. Figure 2a) but also including characteristics of the PZD-FT damper for other voltages within the range of 0–150 V (e.g., 60 V). The intermediate values of the damping force lie between the curves representing no control voltage and the maximum control voltage, and they are obtained for different voltage values. Knowing the desired force T and the rate of change in suspension deflection (and thus the relative velocity of the damper), it is possible to determine the supply voltage from the acceptable range Ω, as shown in Figure 5 (red arrows).

A summary of the suspension control algorithm operation in the form of a block diagram, including the procedure for determining the control voltage for the PZD-FT dampers, is schematically shown in Figure 5. The diagram also illustrates the connections with the sensors and actuators required for its proper functioning.

It is worth noting that the analysis of the situations shown in Figure 4 reveals that, with the use of this control algorithm, it is desirable for the PZD-FT damper to be capable of achieving damping forces in the range of $T\in \left[-S,+S\right]$. This would allow for the realization of damping forces of T=0 or T=S, depending on the needs, enabling the algorithm’s objectives to be more fully realized and most likely increasing the algorithm’s effectiveness. With this type of control, it is also evident that following the path of existing designs may provide benefits (e.g., cost reduction), but also inherits its drawbacks. In the case discussed, basing the PZD-FT damper on the OEM design results in the inability to achieve a symmetric characteristic, which would better meet the requirements of generating a damping force equal to the spring force resulting from the suspension deflection, i.e., T=S. Original solutions have relatively high minimum damping forces that depend on the baseline gap value in the bottom valve.

In the proposed simplified algorithm discussed above, the key to its proper operation is the identification of the suspension parameters for each wheel and its static deflection. The construction of a numerical model for the entire vehicle is unnecessary—a model of a single wheel, known as the quarter-vehicle model, is sufficient.

The presented approach is a simplification based on the experience gained from using more complex and accurate algorithms based on a full-vehicle model [23]. In such cases, it is necessary to identify the parameters of the complete vehicle model and all suspension components. For example, such a model might consist of a rigid plate representing the vehicle body and the suspension of all its wheels. It is also essential to consider the stiffness and damping provided by the tires in the model. To determine these forces, it is necessary to equip the vehicle’s wheels with additional sensors, such as acceleration sensors, to calculate the forces exerted by the wheels on the vehicle suspension. This is not an easy task, and the costs of building such a suspension system increase significantly.

In a more complex algorithm, the control signal values can be determined by optimizing the objective function W, which defines the variation in the wheel load on the road surface. In a way, this algorithm implements a different strategy, namely, the safety criterion. The function W can be described by the following relationship:

(3) $$W=\frac{1}{Q_{st}}\sqrt{\sum_{i=1}^{4}{\left(S_{i}+T_{i}+F_{si}\right)}^{2}},$$

where:

- $Q_{st}$ is the static load caused by the vehicle’s mass, load, and passengers;
- $S_{i}$ is the spring force of the vehicle suspension at the i-th wheel, corresponding to the suspension deflection caused by the static load $Q_{st}$;
- $T_{i}$ is the damping force realized in the damper of the i-th wheel;
- $F_{si}$ is the inertial force of the suspension components and the wheel, which represent the unsprung mass.

Both in the simplified and more complex control algorithms, it is necessary to calculate the damping forces for the dampers of each wheel as a result of the calculations. It is essential to solve the optimization problem, which may involve minimizing the value of the argument under the square root in Equation (3), and can be presented as follows:

(4) $$T_{w}=\underset{T_{i}\in \Omega \left(V_{i}\right)}{\arg\min}\;\left\{\sum_{i=1}^{4}{\left(S_{i}+T_{i}+F_{si}\right)}^{2}\right\},$$

The solution to this problem allows for determining the damping forces that should be applied by the dampers of each wheel, i.e., $T_{w}=\left[T_{1},\ldots ,T_{4}\right]$, and based on the characteristic presented in Figure 2, it is possible to select the appropriate control signal voltages.

Although the control algorithm based on Equation (4) seems advantageous, it is important to consider that its proper functioning also requires a more advanced measurement and control system and appropriate damper characteristics. Specifically, it requires the determination of all the forces in all four wheels of the vehicle suspensions, which is hardly realizable in practice.

Thus, the attention was focused on the development of the simplified control algorithm, which is derived under the following key assumptions:

1. The front wheels are assumed to operate independently, enabling the use of a single-wheel model. This aligns with the tested Ford Transit’s front suspension design, which employs independent control arms to isolate road irregularities.
2. The objective function W, defined by Equation (3), pertains to one wheel only. Summation over all four wheels is omitted due to the lack of sensors mounted in the suspension of the rear axle. Such an approach prioritizes control system simplicity and control algorithm computational efficiency.
3. The wheel inertia, tire stiffness, and damping are omitted under the assumption of consistent wheel–road contact, valid for typical driving scenarios. This allows for focusing on the feasibility of using PZD-FT dampers to control the sprung mass of the vehicle using a few relatively simple and cheap sensors.
4. The suspension inertial force ($F_{si}$), representing the unsprung mass dynamics, is excluded to simplify the optimization problem (Equation (4)).
5. The resulting formulation transforms the safety criterion (minimizing vertical force variations) into an approximate problem of minimizing vertical accelerations at selected locations.

The limitations of the proposed control approach arise directly from the above assumptions. The developed algorithm sacrifices rigorous mathematical optimality in order to ensure computational efficiency and tractability. By neglecting full-vehicle dynamics and deriving control independently for each wheel, the results should be considered as approximate rather than strictly optimal. Nevertheless, the method should perform effectively in scenarios with symmetric wheel excitations, such as straight-line driving on moderately rough roads, where consistent wheel–road contact is maintained. On the other hand, its performance may degrade under asymmetric conditions, such as extreme road roughness or dynamic maneuvers (e.g., hard braking, cornering), where cross-wheel coupling or unsprung mass dynamics become significant. Although wheel detachment was not observed experimentally, the theoretical exclusion of unsprung mass dynamics could lead to inaccuracies in extreme scenarios, such as high-frequency kinematic excitation from road irregularities.

Furthermore, the algorithm’s efficacy depends critically on the hydraulic damper’s mechanical characteristics. For optimal performance, the damper must exhibit rapid response times and a wide force range, enabling precise control across varying road conditions. Mechanical limitations, such as delayed valve actuation or restricted force resolution, could constrain real-world applicability.

Despite these limitations, the algorithm offers significant practical advantages. Its universality lies in its compatibility with any vehicle featuring independent suspension and semi-active dampers. By leveraging standard hydro-pneumatic components and piezoelectric valves, and requiring only suspension deflection as input, the design avoids complex sensor networks or full-vehicle models. This simplicity ensures cost effectiveness, reduced maintenance, and ease of integration into mass-producible systems. The experimental validation on the Ford Transit demonstrated stable performance under diverse conditions, confirming robustness in typical urban driving scenarios. While the solution is suboptimal in a strict mathematical sense, the focus on minimizing vertical accelerations—a proxy for ride safety and comfort—proved to be empirically viable.

In conclusion, the simplifications were deliberately chosen to balance theoretical rigor with real-world feasibility. The algorithm prioritizes practicality, providing a foundation for deployment in common driving conditions while acknowledging its boundaries in extreme or asymmetric scenarios. Future work will explore hierarchical control architectures to address full-vehicle dynamics, but the current approach represents a pragmatic compromise between performance, cost, and computational complexity. At this stage of investigations, this underscores the method’s validity, reliability, and relevance to the automotive industry’s pursuit of efficient, scalable suspension solutions.

## 4. Sensors and Measurement System

As indicated in the previous section, the simplified control algorithm requires knowledge of the forces in the suspension’s elastic elements. Therefore, an essential component of the control system is the sensors, which are required to identify the actual state of the suspension and corresponding response of the vehicle’s body. For this reason, the electronic acquisition system used in the Ford Transit vehicle had to enable the measurement of physical quantities required for the correct operation and verification of the control algorithm (or diagnostic functions of electronic components related to PZD-FT damper control):

- Longitudinal speed and displacement of the vehicle body—signals were measured using a non-contact vehicle displacement sensor, Correvit-L by DATRON (Winturthur, Switzerland);
- Front left and right wheel suspension deflection—signals were measured using displacement sensors PSx by PELTRON (Warsaw, Poland), which operate based on the differential transformer principle—LVDT;
- Vertical, lateral, and longitudinal accelerations of the vehicle body—signals were measured using sensors by HOTTINGER (Darmstadt, Germany);
- Feedback voltages controlling the piezoelectric valves of the dampers—signals were obtained via digital-to-analog converters in the measurement system and the PiezoAMP amplifier (Warsaw, Poland) controlling the APA-120L actuators;
- Three components of the rotational velocity of the vehicle body—measurements were taken using piezoelectric gyroscope sensors by MURATA (Nagaokakyo, Japan);
- Brake pedal pressure signal—information was obtained from a contact sensor.

**Figure 6:**
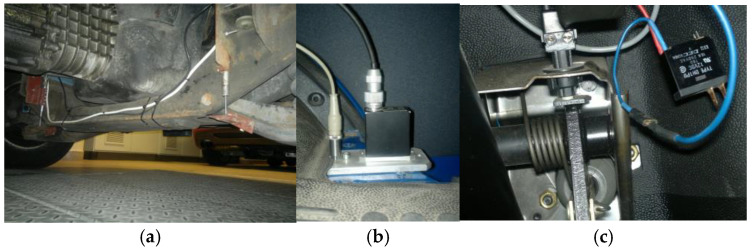
A view of selected sensors used for controlling the PZD-FT dampers in the Ford Transit vehicle. From left to right: (**a**) suspension deflection measurement, (**b**) vertical acceleration measurement, (**c**) wheel arch and passenger seat and brake pedal pressure measurement.

The sensors for physical quantities, actuators, and the electronic system require proper placement and positioning within the vehicle’s structure. A view of the Ford Transit vehicle equipped with the chosen discussed sensors is shown in Figure 6.

**Figure 7:**
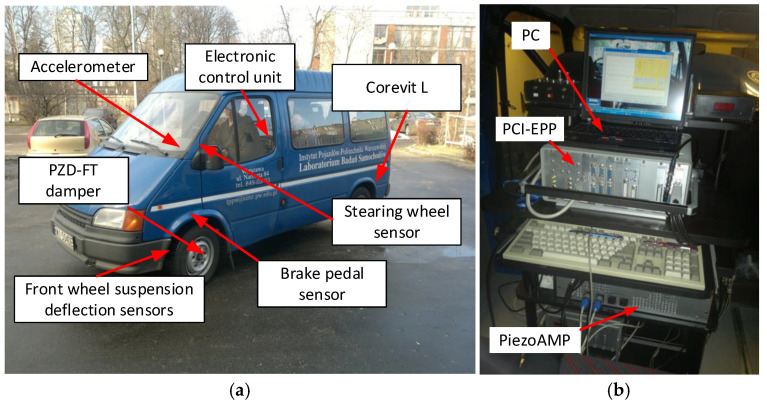
Schematic view: (**a**) suspension control system components for Ford Transit Vehicle with PZD-FT, (**b**) data acquisition system with electronic damper control unit PZD-FT.

The schematic locations of the individual elements of the measurement system for the Ford Transit vehicle are indicated. An overview of the measurement and control system along with all its components is shown in Figure 7. In Figure 7b, a PC is shown used as the data acquisition system for described sensors, operating with sampling 1000 Hz and the PCI-EPP electronic control unit (Warsaw, Poland) used for controlling the PZD-FT dampers and supplying sensors. Also visible is the PiezoAMP amplifier, which is used to power the APA-120L actuators—it is also responsible for converting the control signal from the PCI-EPP system (−1 V to 7.5 V) into the appropriate voltage required for the piezoelectric components (−20 V to 150 V). To reduce the energy consumption, it includes the capability to store recovered electrical energy from actuators.

The sensors for the aforementioned physical quantities enable the identification of events by observing vehicle behavior during various maneuvers or driving conditions, such as braking, accelerating, cornering, or traveling over road irregularities. In particular, these measured parameters also allow for determining the position of the vehicle body relative to the wheels. This is facilitated by suspension deflection sensors and acceleration sensors. The measurement of these signals is crucial for calculating and estimating the forces acting within the vehicle’s suspension system at any given moment, as well as for measuring the accelerations of the vehicle body. Knowledge of these forces is essential for determining the optimal control values for the PZD-FT dampers, ensuring the fulfillment of the adopted optimization criterion.

## 5. Road Tests and Results

**Figure 8:**
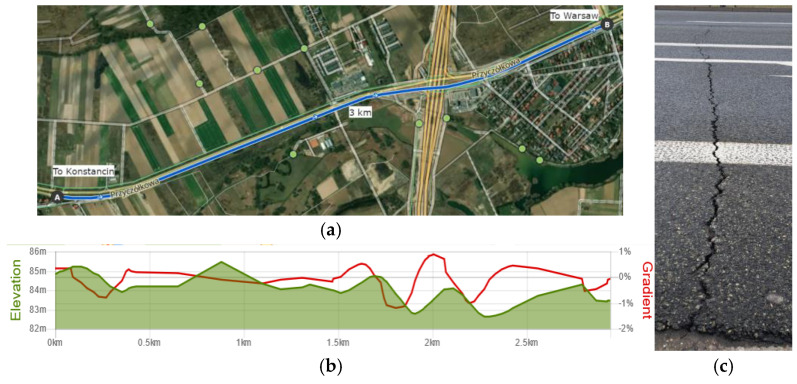
Views of (**a**) the road section between Warsaw and Konstancin on which the road tests were conducted: 52.13446, 21.09989 and 52.15963, 21.08701 (https://www.komoot.com (accessed on 5 February 2025)); (**b**) the height and slope profile (form Konstancin to Warsaw) (https://www.mapometer.com (accessed on 5 February 2025)); (**c**) the exemplary crack at the road surface.

Road tests of the laboratory vehicle Ford Transit were conducted on an approximately 3 km section of a dual carriageway with two lanes in each direction, located between Warsaw and Konstancin—see Figure 8a. This is a typical suburban road of the second category. The road is almost straight, with only three slight curves ranging from 15 to 25 degrees. It is situated at an elevation of approximately 83–87 m above sea level, and its profile is shown in Figure 8b. The road’s gradient is minimal, generally not exceeding 2.5–3%. Therefore, factors related to the road, aside from its unevenness, have a limited impact on the vehicle’s body dynamics. Limited drainage in low-lying sections near the road leads to water pooling and asphalt erosion. As shown in Figure 8c, local road surface damage is visible, recurring cyclically along the length of the road—which suggests moderate roughness (the value of the International Roughness Index, IRI, is about 4.0 m/km).

However, it should be noted that the detailed profile of road roughness was not measured because the control algorithm used did not require it in preliminary investigations. Additionally, measuring road roughness is quite challenging as it requires highly accurate reference measurements over a large area, and such measurement equipment was not available during the study. It is also worth noting that the road surface towards Warsaw visually has fewer such types of damage. This is also visible in the experimental results presented later in this section.

Road tests were conducted and repeated multiple times at constant speeds of 45, 60, and 90 km/h in two modes:

- **Mode 1**: Testing the vehicle equipped with passive suspension (fixed damping) using the factory-installed shock absorbers.
- **Mode 2**: Testing the vehicle equipped with suspension featuring PZD-FT-controlled dampers and describing the simplified algorithm of control.

The purpose of the tests in Mode 1 was to assess the performance of the original suspension system, which served as a reference point for the tests conducted in Mode 2. During the Mode 2 tests, additional evaluations were performed with various gain adjustments for the damper control signal generation. This adjustment affected the rate of increase in the control signal voltage supplied to the dampers, and, consequently, the rate of change in damping forces. The tests in both modes were conducted at constant vehicle speeds of 45, 60, and 90 km.

To evaluate the effectiveness and efficiency of the developed vehicle suspension system, the recommendations from an older edition of the ISO-2631:1985 standard [53] were utilized. Although a newer edition, ISO-2631:1997 [54], is available, the older edition allows for determining exposure times for three vibration perception categories—reduced comfort (called further comfort), fatigue decreased proficiency (annoyance), and health and safety (harmfulness)—which is valuable in the context of vehicles. Additionally, the use of this version of the standard allows for considering not only the total vibration energy measure but also the impact of acceleration impulses at a given frequency on limiting comfort in the vehicle. This approach was also motivated by the availability of software tailored for this type of analysis (PCI-EPP ver. 04.15/6), and by the fact that measurement results based on this standard are still considered reliable [4].

**Figure 9:**
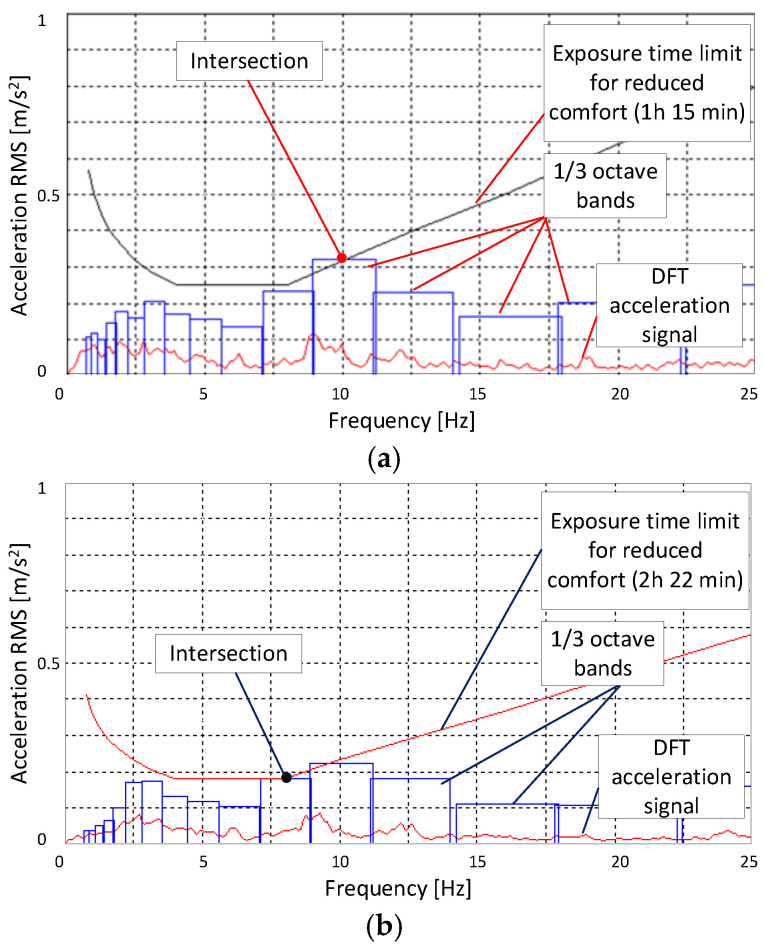
Sample measurements of controlled suspension at V=45 km/h: amplitude spectrum and exposure time boundary of reduced comfort: (**a**) Warsaw–Konstancin; (**b**) Konstancin–Warsaw.

**Figure 10:**
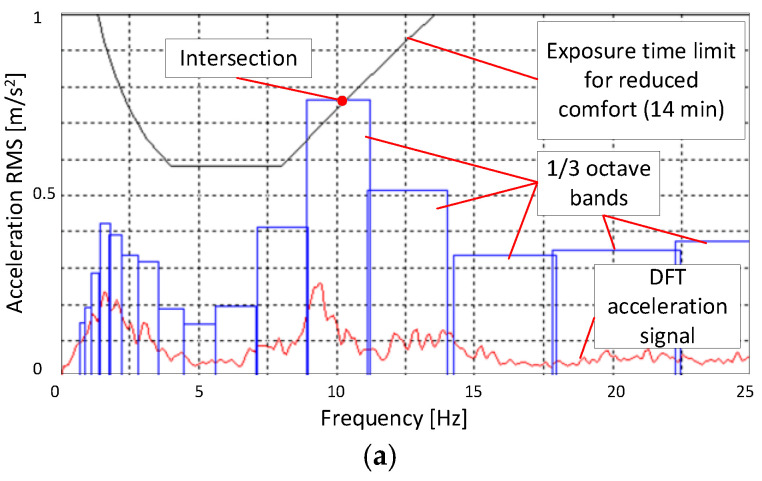

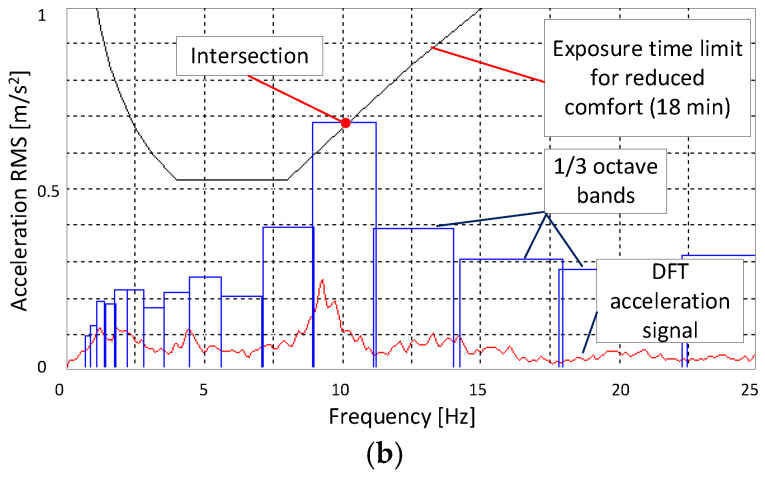
Sample measurements of controlled suspension at V=90 km/h: amplitude spectrum and exposure time boundary of reduced comfort: (**a**) Warsaw–Konstancin; (**b**) Konstancin–Warsaw.

According to the used standard, the analyzed vibration acceleration signal first had to be differentiated with respect to time. The Discrete Amplitude Spectrum (DFT) signal can be achieved by transforming the whole time history signal of a test run to the frequency domain using a Fast Fourier Transform (FFT). The next, frequency spectrum 0.8–80 Hz is divided into 1/3 octave bands. The RMS value of accelerations is evaluated separately for each 1/3 octave band defined in the standard. After that, each RMS value is compared by finding the intersection of a center point of an acceleration bar (cf. Figure 9 and Figure 10) with an exposure line with given limits in the form of so-called exposure time boundaries’ lines for each evaluated category: comfort, annoyance, or harmfulness.

Figure 9 and Figure 10 present selected road test results in the form of amplitude spectra for vertical acceleration signals and the corresponding 1/3 octave band bar spectra calculated for the test road segment at vehicle speeds of 45 and 90 km/h. It is evident that the most significant impact on the exposure time for the tested vehicle comes from accelerations in the frequency range of 0.8–15 Hz.

The mentioned amplitude spectrum enables the determination of permissible exposure times for three vibration perception categories—comfort, annoyance, and harmfulness—based on the boundaries defined in the ISO 2631:1985 standard. This allows for evaluating the effectiveness of damping control in the developed semi-active suspension system of the Ford Transit vehicle. Table 1 and Table 2 present the exposure time results according to ISO 2631:1985 for the three categories, comfort, annoyance, and harmfulness, for two opposite driving directions (separate road lanes).

The smaller the values of exposure time achieved, the worse and more uncomfortable the travel conditions are for the vehicle users. Exceeding the exposure times associated with harmful effects can result in the development of vibration disease, which is nowadays increasingly being diagnosed.

The analysis of the results presented in Figure 9a,b shows that on the section towards Warsaw (with fewer irregularities), the frequency of accelerations is dominant in the range of 2–3 Hz and 8–10 Hz. On the section towards Konstancin, accelerations in the frequency range of 8–13 Hz become more dominant, which limits the exposure time. While frequencies in the range of 2–3 Hz can be associated with body vibrations resulting from road irregularities, frequencies of 8–13 Hz are more likely related to vibrations transmitted from the wheel and suspension components. This becomes even more apparent at a speed of 90 km/h, see Figure 10a,b, where kinematic excitations from road irregularities have a higher frequency. As a result, body accelerations in the frequency range of 10 Hz become dominant and increase from the range of 0.2–0.3 to 0.69–0.78 m/s^2^. Accelerations in this range significantly affect the reduction in exposure time at a speed of 90 km/h.

Table 1 presents the averaged results from multiple trips taken on the section from Warsaw to Konstancin, where greater road irregularities were observed. Meanwhile, Table 2 contains the results for the section from Konstancin to Warsaw.

The analysis of the results presented in the tables above indicates that the vehicle body acceleration with passive dampers on both test routes reaches similar values. Similarly, the obtained exposure times are very similar at a speed of 45 km/h, regardless of the direction of travel. The results for the road with a better surface compared to a worse one (direction from Konstancin to Warsaw) differ by about 2% to 25%, respectively, for velocities of 45 and 90 km/h. It is also visible that road irregularities, which were emphasized, begin to play a larger role as the speed increases to 60 and 90 km/h. As the speed increases, the exposure time decreases sharply, and the exposure times on both sections start to differ more significantly.

However, a comparison of the effects of passive suspension and the controlled suspension system with PZD-FT dampers indicates that speed also plays a significant role and impacts the quality of the results. When applying the semi-active suspension at low vehicle speeds, such as 45 km/h, the exposure time in some categories increased by as much as 29–136%, depending on the direction of travel. At a speed of 60 km/h, the differences were around 70–88%, while effectiveness sharply decreases as speed increases to 90 km/h. At this speed, exposure times were either increased or even decreased by 6–11%, depending on the compared category.

**Figure 11:**
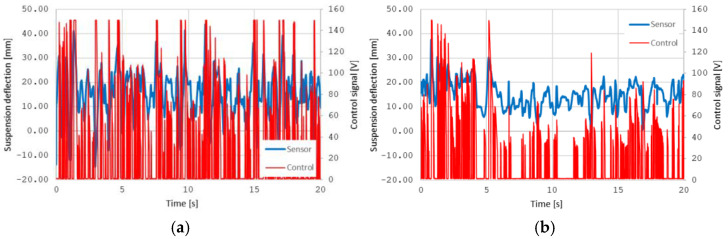

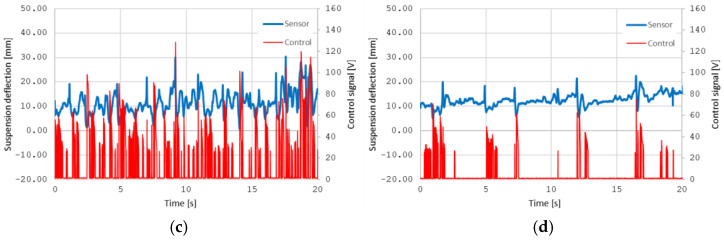
Exemplary results of the suspension deflection of the left wheel and the PiezoAMP control signal voltage of PZD-FT dampers at a speed of (**a**) 90 km/h to Konstancin, (**b**) 45 km/h to Konstancin, (**c**) 90 km/h to Warsaw, and (**d**) 45 km/h to Warsaw.

The comparison of the results presented in Figure 9 and Figure 10 also shows differences in exposure time for different speeds as well as for the direction of travel to Konstancin or Warsaw. In Figure 11, it is evident that the suspension deflections at the same speed are significantly greater on the road towards Konstancin. In the direction of Warsaw, the suspension deflections are smaller, and it is noticeable that at a speed of around 45 km/h, the control algorithm adjusts the PZD-FT damper voltage only for larger, recurring road irregularities. The shape of these irregularities can also be partially found in Figure 11c, although it is evident that the same road irregularities at higher speeds cause greater suspension operating speeds.

The observed limitations in the control system’s effectiveness at higher speeds, particularly 90 km/h, stem from the intensified dynamic forces acting on the vehicle. At elevated speeds, road irregularities are encountered at a faster rate, leading to shorter excitation intervals and higher-frequency vibrations. The semi-active PZD-FT dampers, while capable of adjusting damping forces (damping coefficient within a range of 1500–9000 Ns/m and dimensionless damping coefficients of about 0.12–0.73) at lower speeds (45–60 km/h), encounter physical limitations under these harsher conditions. As demonstrated in Figure 11a, when there are quite moderate irregularities, the dampers can also frequently operate at their saturation limits ($T_{min}$ and $T_{max}$), where the required damping force to counteract rapid excitations exceeds the adjustable range of the PZD-FT damper. It should be noted that $T_{min}$ saturation does not imply a damping force of zero during damper operation. Any realized force means that problem defined by Equation (1) is indeed solved, but this solution is not optimal if the damper could achieve smaller forces at higher speeds. This saturation can restrict their ability to isolate vibrations effectively, resulting in prolonged exposure times. Furthermore, the simplified control algorithm introduces a phase lag, as it lacks the bandwidth to respond to rapid changes in road input, leading to a mismatch between damper action and excitation frequency.

The coupling between road unevenness and suspension dynamics plays a critical role in the system’s behavior. Road roughness profiles impose excitations that scale with vehicle speed v—for example, a sinusoidal road irregularity with a wavelength λ generates a forcing frequency proportional to v/λ. At 90 km/h, common road wavelengths (2–5 m) correspond to excitation frequencies of 5–12.5 Hz, which overlap with the natural frequency range of the vehicle’s sprung mass (1–15 Hz). This overlap induces resonance effects, amplifying vertical oscillations, particularly in the rear suspension. The rear solid axle, equipped with passive dampers, cannot adapt dynamically to dissipate this energy, unlike the semi-active front dampers. The rigid rear suspension’s fixed damping coefficient becomes suboptimal at high speeds, allowing uncompensated pitch and bounce motions to propagate through the vehicle body.

The asymmetric configuration of the suspension system—semi-active dampers on the front axle and passive dampers on the rear—introduces dynamic imbalances. While the front dampers adjust to road inputs, the rear passive dampers lag in energy dissipation, creating coupled oscillation modes that the control system cannot fully mitigate. This asymmetry allows vibrations from the rear axle to influence the vehicle body, further increasing exposure time. Additionally, the control algorithm’s neglect of wheel–tire dynamics, such as tire elasticity and unsprung mass effects, limits its accuracy. At high speeds, tire-hop modes (10–20 Hz) interact with suspension vibrations, altering load transfer and reducing tire–road contact. These unmodeled dynamics amplify body oscillations, which the current control strategy fails to address.

To address these challenges, future work should focus on developing a multi-input control algorithm that coordinates all four dampers and incorporates tire dynamics. Expanding the damping force range of the semi-active dampers and improving the controller’s bandwidth could reduce saturation effects and phase lag. Validation against standardized road profiles, such as ISO random road inputs, would also help quantify speed-dependent coupling mechanisms more rigorously.

An additional influence is related to delay in the response time of the suspension measurement and control system. For example, at a constant speed of 45 km/h and a response time of 9–10 ms for the PZD-FT dampers, the vehicle covers a distance of about 12.5 cm. At a speed of 90 km/h, this distance increases to 25 cm. An analysis of the static contact between the tire and the road surface indicates that the contact patch length (the length of the contact area) of the laboratory vehicle’s tire with the road is approximately 17 cm at standard tire pressure. This means that for more than 10 ms, a typical road irregularity remains in contact with the tire and can directly affect the suspension deflection and its measurement with the used sensor. Therefore, it is likely that for speeds of 45 and 60 km/h, satisfactory results were achieved, as the measured suspension deflection was directly linked to the road irregularities. At higher speeds, however, the suspension deflection measurement corresponds to different road irregularities. Therefore, the issue of detecting road irregularities at an appropriate distance ahead of the vehicle is crucial. This distance should be longer the higher the vehicle’s speed is, and the slower the controlled dampers are in their response. Manufacturers are continuously finding newer ways to address this challenge [55,56].

Nevertheless, the results obtained indicate that even the application of a controlled suspension in a semi-active system in front wheel suspension can decisively affect its performance efficiency. Example results showed an increase in exposure time by more than 150% of the initial value. One can easily imagine the impact of combining the discussed suspension system with additional sensors for analyzing road irregularities ahead of the vehicle. The effectiveness of such a system could significantly increase even further.

In summary, the control algorithm proposed in this paper is characterized by the following unique features that distinguish it from aforementioned control methods:

- The proposed method does not require the preliminary development of a detailed mathematical model of an entire vehicle or half-vehicle. As a result, it is independent of modeling accuracy and variations in model parameters over time.
- The method relies solely on real-time suspension deflection measurements, which can be easily and precisely obtained using simple standard displacement sensors. This eliminates the need for a complex sensor system combining various measurement techniques.
- The algorithm employs a simple semi-heuristic analytical approach to compute the required control. This allows us to avoid complex numerical computations and minimizes potential control delays.
- The proposed system incorporates an additional bypass and piezoelectric valve, which can be integrated into most standard hydraulic dampers commonly used in vehicles’ suspensions.
- From a practical perspective, the entire system is highly reliable and robust, with relatively low costs of construction, operation, and maintenance.

## 6. Conclusions

This paper presents the experimental results for the semi-active suspension system of a vehicle equipped with PZD-FT-controlled dampers with the piezoelectric valve. The combination of relatively good damper properties with simple control algorithms allows for a quick response of the suspension to road irregularities. The road tests conducted demonstrated good suspension performance at speeds up to approximately 60 km/h. Up to this speed, a significant improvement in the values of the applied comfort measurement criteria was achieved. The results of the performed study show that with the fast-reacting PZD-FT dampers, significant improvements in ride comfort can be achieved at typical urban driving speeds without the need for expensive sensors and road irregularity recognition systems ahead of the vehicle. However, it should be noted that the use of such sensors could enhance the system’s performance, particularly at higher speeds.

The use of a simplified control algorithm helps to avoid some of the issues associated with advanced models and their longer operation times. The obtained results also indicate that for the correct operation of the proposed semi-active vehicle suspension, it is sufficient to use only two suspension displacement sensors—one for each wheel. This approach ensures a low computational cost, which is significantly higher in the case of more advanced algorithms. The higher cost is associated with longer computation times, which can result in valuable time potentially being wasted as the vehicle travels additional distances and encounters further road irregularities. However, it is important to note that with current computing power in automotive computers, such delays will not be as significant as the delay caused by the response time of controlled dampers.

The analysis of the obtained results indicates that the effectiveness of the developed suspension could most likely be improved by using a damper with a slightly different characteristic, one that allows for damping forces closer to zero. Additionally, further reducing the damper’s response time should enable the system to operate more effectively at higher speeds, as it would be possible to shorten the section of road that needs to be detected by the sensor system.

It is also worth recalling that the discussed semi-active suspension was built based on controlled dampers placed in the front axle suspension. Numerical analyses presented in many research papers indicate that using controlled dampers on all wheels would likely lead to an even greater improvement in the effectiveness of the developed suspension.

The results obtained also suggest that it may be beneficial to incorporate suspension models that consider the length of the wheel’s contact patch with the road surface when developing more complex control algorithms. Interestingly, the length of this contact patch can also be influenced by the size of the tires and tire pressure, which could be an interesting design parameter to consider when developing a new semi-active vehicle suspension system.

Further research should focus on the continuous application of piezoelectric actuators, primarily of the APA type, which, due to their short response time of less than 10 ms, enable precise control of hydraulic damper operation and significantly enhance vehicle dynamics. In addition to control strategies tailored to meet comfort criteria based on maximum acceleration values, more complex strategies addressing safety criteria emphasizing the variability of forces acting on the wheels will be developed. For relatively high-speed scenarios, where conventional wheel acceleration measurements may prove to be insufficient, it is envisioned to implement a predictive measurement of road roughness with the use of a radar sensor. The proposed control strategies will be evaluated under diverse road conditions, encompassing varying inclinations, curvatures, and uneven surfaces affecting both wheels, where distinct control algorithms may be required. Subsequent studies will further assess the efficacy of the developed algorithms in addressing dynamic scenarios including vehicle acceleration, braking, and cornering.

## Figures and Tables

**Table 1:** The comparison of exposure times for passive and controlled shock absorber suspension on the test section of the Warsaw–Konstancin route.

Velocity [km/h]	Type of Suspension and Exposure (Warsaw -> Konstancin)					
	Passive			Semi-Active PZD-FT		
	Comfort	Annoyance	Harmfulness	Comfort	Annoyance	Harmfulness
45	0 h 58′	8 h 37′	24 h 00′	1 h 15′	11 h 09′	24 h 00′
60	0 h 20′	2 h 58′	11 h 48′	0 h 33′	5 h 01′	20 h 07′
90	0 h 15′	2 h 08′	8 h 30′	0 h 14′	2 h 22′	9 h 28′

**Table 2:** The comparison of exposure times for passive and controlled shock absorber suspension on the test section of the Konstancin–Warsaw route.

Velocity [km/h]	Type of Suspension and Exposure (Konstancin -> Warsaw)					
	Passive			Semi-Active PZD-FT		
	Comfort	Annoyance	Harmfulness	Comfort	Annoyance	Harmfulness
45	1 h 00′	8 h 56′	24 h 00′	2 h 22′	21 h 17′	24 h 00′
60	0 h 23′	3 h 28′	14 h 50′	0 h 43′	6 h 30′	24 h 00′
90	0 h 19′	2 h 37′	11 h 07′	0 h 18′	2 h 47′	11 h 11′

## Data Availability

The data is available upon request.
